# Relapsing Malaria: A Case Report of Primaquine Resistance

**DOI:** 10.1155/2018/9720823

**Published:** 2018-03-11

**Authors:** Christopher Dijanic, Jillian Nickerson, Sunita Shakya, Amanda Dijanic, Marilyn Fabbri

**Affiliations:** ^1^Northeast Ohio Medical University, Rootstown, OH, USA; ^2^Elmhurst Hospital Center, Elmhurst, NY, USA; ^3^Mount Sinai School of Medicine, New York, NY, USA; ^4^Kent State University, Kent, OH, USA

## Abstract

Primaquine (an 8-aminoquinoline malarial therapy) is the only FDA-approved therapy to treat the hypnozoite stage of *P. vivax*. We think of relapse occurring because of parasitic resistance or poor compliance secondary to drug toxicities. However, in patients with repeated treatment failure, we must consider CYP-450 mutations affecting drug metabolism as an important cause of relapse. A 47-year-old man who travelled to a jungle in Venezuela was diagnosed with *P. falciparum* and *P. vivax* in July 2015. He was treated with seven rounds of primaquine-based therapy in the following year, all resulted in relapse without further exposure to endemic areas. On his eighth presentation, he was found to have CYP-4502D6 mutation that affected the metabolism and activation of primaquine. Thereafter, he was treated without relapse. Primaquine efficacy depends on many factors. Understanding the mechanism responsible for malaria relapse is paramount for successful treatment and reduction in morbidity and mortality. This case illustrates the importance of considering cytochrome mutations that affect drug efficacy in cases of relapsing malaria.

## 1. Background

*Plasmodium vivax* is a common infection that can result in significant morbidity and mortality when not treated [1, 2]. According to recent studies, approximately 2.5 billion people worldwide are at risk for *Plasmodium vivax* infection [3]. Of those at risk, clinical health complications occur in about 100–400 million people [1]. In fact, 10–20% of patients diagnosed with a primary infection of *P. vivax* or *P. falciparum* were determined to have a severe infection, and 10–15% of patients diagnosed did not survive [1]. While *P. vivax* was previously considered a benign infection, recent reports have established that certain populations experience higher rates of severe infection [4]. A study performed in Thailand demonstrated that pregnant women were three times more likely to have malaria than nonpregnant women. Furthermore, those infected during pregnancy were three times more likely to have a miscarriage due to premature birth, stillbirth, and reduced birth weight. They also found that risky environmental exposures combined with lack of health care accessibility greatly increased health risk amongst migrant workers and impoverished populations [3, 4]. According to the United States (U.S.) Centers of Disease Control and Prevention (CDC), severe disease includes patients with cerebral edema, coma, seizures, anemia, thrombocytopenia, circulatory collapse or shock, or vital organ dysfunction such as ARDS, respiratory failure, acute renal failure, splenic rupture, hepatic dysfunction, or jaundice [4]. Thus, successful treatment is essential to prevent complications, especially in high-risk groups.

In addition to the high rate of complications, *P. vivax* gametocytes transmit more effectively to the mosquito vector than *P. falciparum* [3]. Moreover, *P. vivax* is able to transmit to its vector at lower parasitic densities [3]. Therefore, people living in *P. vivax* endemic areas are at high risk for contracting the parasite.

Treatment and eradication of *P. vivax* are complicated by its ability to remain dormant within the host. When dormant, it is difficult to detect, especially in locations without proper lab facilities. Low rates of detection result in low rates of treatment. As a result, *P. vivax* frequently causes recurring episodes during months following a primary infection for up to two years [4]. Extreme heath consequences associated with untreated *P. vivax* demand for successful treatment and cure of the cases.

Currently, primaquine is the only licensed and FDA-approved treatment for *P. vivax*. While studies demonstrate high efficacy associated with the drug, there are several limitations that prevent the eradication of *P. vivax* from endemic regions. For example, it has limited use in certain populations. The oxidative stress produced by drug metabolites causes intravascular hemolysis in individuals with G6PD deficiency [3]. This represents an enormous challenge to primaquine use as approximately 500 million people worldwide suffer from G6PD deficiency [1]. Additionally, primaquine is not used in pregnant women due to concern for fetal hemolysis if the fetus is G6PD positive [3]. In addition to hemolysis, primaquine requires long courses of therapy for effective treatment, thereby lowering compliance. Commonly used pharmacologic agents, such as antiarrhythmic and antihypertensives, cause drug-drug interactions reducing the efficacy of primaquine [5]. Additionally, parasitic resistance limits efficacy [3].

The aforementioned limitations of primaquine treatment have long been known and understood. However, recent studies have also established the importance of *CYP2D6* mutations on primaquine efficacy. Studies performed on mice with knockout genes encoding for the *CYP2D6* enzyme demonstrated no primaquine activity at therapeutic doses [3]. Primaquine, an 8-aminoquinoline, is a prodrug requiring hydroxylation by the *CYP2D6* enzyme within hepatocytes to transform into the active form [5]. Humans exhibit genetic variations affecting the *CYP2D6* gene rendering them null, poor, intermediate, or fast metabolizers. Patients with lower levels of *CYP2D6* metabolization experience recurrent malarial episodes regardless of proper treatment regimens with primaquine [5].

## 2. Case Presentation

A 47-year-old man diagnosed with malaria in the summer of 2015 presented in May 2016 with a five-day history of symptoms. He noted that five days prior to presentation, he started feeling generalized weakness. Four days before presenting, he felt lightheaded and had one episode of nonbilious, nonbloody vomiting, later developing right upper quadrant pain. He also endorsed several bouts of nonbloody diarrhea for the three days prior to arrival with associated fevers up to 102°F. On review of systems, the patient endorsed frontal headaches and dark urine. He denied any recent travel or sick contacts.

However, the patient's symptoms had actually begun almost a year before. During July of 2015, after the patient spent twenty days in a Venezuelan jungle, he had a seizure and was taken to local health clinic where he was treated with an unknown regimen of antimalarial pills for fourteen days. After a brief period of resolution, the patient experienced his second clinical episode and went to a rural town where he was again treated with an unknown regimen of antimalarial medications.

After returning to the U.S., during October of 2015, the patient remained asymptomatic until he experienced a third episode of malarial symptoms for which he was treated in Miami, Florida (though unable to recall/establish precise regimen). On November of 2015, the patient endorsed a fourth clinical episode with the same symptoms including fever, myalgia, nausea, vomiting, diarrhea, and right upper quadrant pain. At that time, he was admitted to Elmhurst Hospital in Queens, New York. He was diagnosed by blood smear analysis with both *P. falciparum* and *P. vivax* during that hospitalization. He was treated with quinine for five days, doxycycline for seven days, and primaquine for fourteen days.

He was readmitted and retreated at Elmhurst Hospital for malaria during both January and February of 2016 with the same medication regimen stated previously; but during February, the dose of primaquine was doubled and he was enrolled in directly observed therapy (DOT) in Elmhurst Hospital ID Clinic to ensure compliance. After completion of his last regimen of primaquine on March 10, 2016, he reported to be asymptomatic.

On May 8, 2016, he was readmitted to Elmhurst Hospital for recurrent malaria (at which point he was treated by study authors). This was his seventh episode of malaria over the course of one year. By his seventh presentation, he had completed an approved treatment regimen, endorsed compliance, and had no risk for reexposure. The CDC was then contacted by study authors via CDC Malaria Hotline, given multiple relapses, at which point it was suggested genotyping be sent for *CYP2D6*. When tested for the *CYP2D6* allele during his May 8th admission, he was found to have a ^∗^4/^∗^5 allele variation, which corresponds to a poor metabolizer. There are numerous alleles rendering *CYP2D6* inactive, and ^∗^4/^∗^5 are among those “null alleles” [6].

DNA analysis of the cytochrome *P4502D6* gene was performed at North Shore-LIJ Core Laboratories located in Lake Success, NY. Testing was completed on the Tm Bioscience/Luminex Universal Array platform using primer extension chemistry. Multiplex PCR-amplified fragments containing alleles aforementioned and the primer extension then generated a biotin-labeled product that hybridized to complementary bead-immobilized probes to permit flow-sorted detection of both normal and variant sequences. This testing is available through the U.S. Centers for Disease Control and Prevention (CDC) by contacting the CDC Malaria Hotline, information for which is readily available online.

Using the CDC guideline, the patient was started on chloroquine for 48 hours and discharged. After completion of the chloroquine regimen, he was placed on weekly chloroquine prophylaxis for 1-2 years. Since completion of the aforementioned treatment, he has remained malaria free.

## 3. Interpretation

The patient suffered an attack of *P. vivax* after he travelled to an endemic area. He was treated twice with unknown medications while abroad. However, upon his return, he was treated with four full courses of appropriate medications. After failure of resolution and continued relapses, a genetic screen was ordered, and it was determined that the patient had a mutation of the *CYP2D6* allele rendering him a “poor metabolizer,” thereby explaining his treatment failure.

Previous studies established the pivotal role of *CYP2D6* hepatocyte enzyme on primaquine metabolism and *P. vivax* eradication. Those who were intermediate and poor metabolizers were considered for alternate therapy with chloroquine, artemether, or lumefantrine [3]. Our case validates previous claims on the crucial role that genetic variations of the *CYP450* have on proper therapy. After numerous failed treatments and a genetic screen, second-line therapy was initiated successfully eradicating the *P. vivax* infection.

## 4. Conclusions

The findings of this case represent therapeutic failure of primaquine due to an allele mutation of *CYP2D6* rendering the individual a poor metabolizer. While primaquine remains the therapeutic treatment for *P. vivax*, the lack of alternative treatments leaves a portion of the population at risk for life-threatening complications of untreated infections. There is need for future studies that will help to verify the utility of second-line therapies in the case of primaquine failure, especially in those with *CYP2D6* mutations.
